# PD67 Real-World Evidence To Inform Reflexive Practice And Create Value In Lung Cancer Care

**DOI:** 10.1017/S0266462324003246

**Published:** 2025-01-07

**Authors:** Gino Boily, Aude-Christine Guédon, Godwill Abiala, Samia Qureshi, Kossi Thomas Golo, Ferdaous Roussafi, Camille Lehuédé, Julie Lanthier, Kahina Soltana, Frédéric Kuzminski, Erin Strumpf, Jim Boulanger, Élisabeth Pagé, Catherine Truchon

## Abstract

**Introduction:**

Health technology assessment (HTA) agencies assess the value of innovative therapies and publish recommendations for practice. However, is publishing HTA products sufficient to generate value in the real world? The objectives of our work were to: (i) determine whether innovative therapies for lung cancer produce the expected results in the real-world setting; and (ii) assess whether recommendations are followed in real-world practice.

**Methods:**

Clinical administrative data were used in this two-phase project. In the first phase, a descriptive portrait of the use of epidermal growth factor receptor tyrosine kinase inhibitors (EGFR-TKIs) for treating lung cancer was produced. Their value was assessed by comparing overall survival of treated patients observed in the province of Québec to the published literature. The second phase focused on the initial evaluation of patients diagnosed with lung cancer and treated first by surgery. The delay between first evidence of cancer and surgery was assessed, and the utilization of 27 healthcare services was analyzed and assessed according to our recommendations (algorithms) for lung cancer management.

**Results:**

From the date the first EGFR-TKI was listed, it took about five years before these drugs were fully integrated into clinical practice. The median overall survival of patients in Québec who used an EGFR-TKI (three indications) was similar to that in most published studies, supporting previous reimbursement decisions. The median delay between first evidence of cancer and surgery was longer than the 60-day consensus target. Utilization of most healthcare services was heterogeneous between regions. Bronchoscopy on its own seemed overused in many regions, whereas non-surgical approaches as a first method for invasive mediastinal evaluation should have been more systematically applied.

**Conclusions:**

At a relatively low cost, real-world evidence can serve as a powerful tool to validate reimbursement decisions and measure the state of clinical practice. By sharing results with stakeholders, it will enable clinical teams to reflect upon their practice and implement local improvement strategies.

